# Behcet’s disease with recurrent thoracic aortic aneurysm combined with femoral artery aneurysm: a case report and literature review

**DOI:** 10.1186/s13019-017-0644-y

**Published:** 2017-09-06

**Authors:** Shi-Huai Zhang, Fu-Xian Zhang

**Affiliations:** 1 Department of General surgery, Fuxing Hospital of Captial Medical University, No. 20 of Fuxingmenwai Street, Xicheng District, Beijing, 100038 China; 2 Department of Vascular surgery, Shijitan Hospital of Captial Medical University, Beijing, 100038 China

**Keywords:** Behcet’s disease, Aneurysms, Corticosteroid therapy

## Abstract

**Background:**

Aneurysm or pseudoaneurysm is the main vascular complication of Behcet’s disease. Most hospitals adopt endovascular treatment.

**Case presentation:**

We report a case of Behcet’s disease with recurrent thoracic aortic aneurysm combined with femoral artery aneurysm. The patient underwent two rounds of endovascular surgery, but developed new aneurysms immediately after surgery. Eventually, the patient died due to rupture of recurrent aneurysm.

**Conclusions:**

For vasculo-Behcet’s disease, we suggest performing the operation during the stable period. At the same time, glucocorticoids could be used with immunosuppressants preoperatively and postoperatively.

## Background

Behcet’s disease (BD) is a vasculitis of unknown origin. BD is rare in Western countries and in the southern hemisphere. It is mainly seen in countries along the Silk Road such as the Middle East and Mediterranean regions. It is also distributed in Central and East Asian countries including Korea, Japan and China [1]. This disease was first described in 1937 by a Turkish dermatologist, Behcet H [2]. BD is a chronic systemic inflammatory disease that involves the mucous membranes, skin, eyes, gastrointestinal tract, joints, vessels and neurologic system. The involvement of the vascular system in BD is called vasculo-Behcet’s disease. The incidence of vascular involvement in patients with Behcet’s disease is 7 to 38% [3]. Vascular involvement can include both arteries and veins, with lesions ranging from arterial occlusions and aneurysms to superficial thrombophlebitis. The majority of patient deaths due to vasculo-Behcet’s disease are related to rupture of the aneurysms. We report a vasculo-Behcet’s disease patient with recurrent thoracic aortic aneurysm combined with femoral artery aneurysm. The patient underwent endovascular surgery. However, the patient eventually died due to rupture of recurrent aneurysm.

## Methods

Ethical statement: This study was approved by the Ethics Committee of Shijitan Hospital, Capital Medical University. Since our case report does not violate the patient’s privacy, informed consent was not necessary.

## Case report

A 30-year-old Chinese woman from Hunan province was admitted to our hospital due to relapsing oral ulcer for 2 years, with progressive chest and back pain for 4 months. In a local hospital, the patient’s CTA examination revealed false aneurysm of the thoracic aorta. Hence, endovascular repair of aortic aneurysm was performed. The back pain was not significantly relieved after the operation, and the aorta CTA examination revealed false aneurysms at the original distal-end of the stent-graft and at the original incision at the femoral artery. Hence, the patient came to our hospital. The patient denied any history of hypertension, diabetes, or drug abuse. The pathergy test result was positive, and laboratory test results revealed a erythrocyte sedimentation rate of 45 mm/h (reference range: 0–15 mm/h) and C-reactive protein of 110.2 mg/L (reference range: 0–10 mg/L). Methylprednisolone was used at 80 mg per day. One week later, the patient’s chest and back pain suddenly aggravated, and this condition could not be relieved even with the use of analgesic drugs. Through CTA, it was inferred that the aneurysm ruptured because the thoracic aortic aneurysm diameter was larger than its previous size. Hence, emergency surgery was performed. Stents were placed at the distal end of the original stent and the inferior segment of the right femoral false aneurysm. The patient was given 40 mg of prednisone and 0.5 mg of colchicine daily after the operation. ESR level was 10 mm/h and CRP was 16.79 mg/L. Furthermore, the chest and back pain of the patient was also relieved after 2 weeks. The patient was discharged and continued to orally take 20 mg of prednisone and 0.5 mg of colchicine daily. However, the patient died due to rupture of recurrent aneurysm 1 month later in a local hospital.

## Discussion

Vasculo-Behcet’s disease involving large arteries can lead to thromboses, occlusions and aneurysms. It is very dangerous when the aneurysm ruptures. The mortality of pulmonary aneurysm rupture is approximately 50% [4]. The reasons for aneurysm rupture are based on two aspects: blood vessel inflammation itself can destroy the arterial wall, and the nutrient vessels of the artery are damaged and aggravate the injury of the arterial wall.

The treatment of vasculo-Behcet’s disease includes medicine and surgery. Corticosteroid therapy and immunosuppressants have been used for BD. Cyclosporine, azathioprine, anti-tumor necrosis factor (TNF) agents, and interferon-α (IFN-α) has begun to revolutionize the treatment of BD [5]. Previously, open surgical repair was the definitive treatment for vascular lesions such as aneurysm in BD patients. However, some studies have reported its recurrence following surgical management in approximately half of the cases. Okada [6] reported their experiences with its recurrence in eight patients with long-term follow-ups. Four cases (50%) required a second operation, and two of these patients underwent a third operation due to recurrence. In order to avoid complications stemming from surgical repair, the endovascular insertion of a stent-graft may be a reasonable alternative [7].

Nowadays, endovascular treatment combined with immunosuppressive drugs has a tendency for vasculo-Behcet’s disease to occur [8]. However, aneurysm recurrence after the operation is also a major problem in vasculo-Behcet’s disease, in which even the puncture site can form a false aneurysm after diagnostic angiogram [9].

The etiology of BD remains unknown, and the pathological basis of BD is peripheral inflammation. Abnormal neutrophil activity is considered to be the main pathogenesis of BD. The reason for aneurysm formation is that endarteritis obliterans is induced by occlusions of the vasa vasora of large and middle arteries [10]. In order to reduce the recurrence of aneurysm and mortality after the operation, the endovascular approach would be feasible, and the operation should be avoided in the active phase of BD [11]. Applied drug control BD in the active phase is the foundation of surgery. Although endovascular procedures are less invasive than open surgery, stents or stent grafts may provoke inflammation, and mechanical irritation could contribute to the recurrence of psedoaneurysm after endovascular treatment. The pre- and postoperative use of immunosuppressants for BD patients to modulate inflammation could thereby reduce the risk of complications. The immunosuppressive regimen is adjusted based on the serial measurement of ESR [12].

We suggest that the time of using immunosuppressants should be adequate. In our case, the patient underwent two rounds of endovascular treatment. However, the patient developed a new aneurysm after each operation. Finally, the patient died due to rupture of aneurysm. Although the patient received a high-dose of steroid and colchicine, the time course of immunosuppressive agents was not adequate. Hence, the result was bad. Apart from critical patients, we suggest that the operation should be performed until the inflammatory markers are diminished. The postoperative application of corticosteroids with immunosuppressants for at least 2 years is effective [13, 14]. The perioperative administration of corticosteroids with immunosuppressants is also effective in decreasing the incidence of recurrent aneurysm [15].

## Conclusion

In conclusion, we suggest that surgical operations including endovascular treatment should be avoided in treating aneurysms of vasculo-Behcet’s disease, except for patients under life-threatening conditions. When scheduling for an operation, we should avoid the active phase of BD, and determine to operate when ESR and CRP are normal. Postoperative glucocorticoid and immunosuppressive drugs needs to be continuously used, and patients should continuously undergo long-term observations and follow-ups.

## Abbreviations

IFN-α: Interferon-α
BD: Behcet disease
TNF: Tumor necrosis factor
